# A participatory protocol for collective decision-making in cultural organisations

**DOI:** 10.12688/openreseurope.21601.1

**Published:** 2025-11-06

**Authors:** Mina Dragouni, Vasilis Avdikos, Martha Michailidou, Dimitris Pettas, Nikolaos Georgantzis

**Affiliations:** 1 Department of Economic and Regional Development, Panteion University of Social and Political Sciences, Athens, Attica, Greece; 2 Department of Communication, Media and Culture, Panteion University of Social and Political Sciences, Athens, Attica, Greece; 3 Burgundy School of Business, Dijon, Bourgogne-Franche-Comté, France

**Keywords:** cultural organisations, participation, participatory decision-making, participatory tools, participatory workshops

## Abstract

Over the last decades, museums and cultural organisations in Europe are increasingly turning towards inclusive and collaborative approaches to engaging with the public. While participatory work has become present in diverse forms and levels across sectoral practice, effective tools and frameworks for embedding participation into governance and everyday decision-making are still relatively scarce. This gap highlights a critical need for experimenting with participatory designs that enable cultural organisations to pursue and practically realise open and democratic modes of management and organisational processes. Along these lines, the contribution of this paper is twofold: First, it opens a window into the functioning of decision-making processes in cultural organisations. Second, it proposes and experimentally tests a protocol for participatory decision-making with cultural organisations in France, Greece and Germany. The protocol involves, first, an “anonymous” stage in which participants engage in a two-step “proposal-then-Borda voting” process. Each participant’s proposed agenda is the ideal combination of traits of a project to be developed by the team. Once all the proposed projects are received and anonymously presented to all participants, a Borda voting rule is applied to select the “winning” project. A brainstorming session follows, where participants publicly discuss the winning project and propose variations or major modifications until a unanimous decision is reached. This protocol was developed and tested in three real-world collective decision-making sessions with members of cultural organisations during participatory workshops in Dijon, Athens and Berlin. The protocol shaped decisions openly and democratically, combining (i) endogenous project agenda formation, which translates individual preferences into sets of alternative solutions, (ii) a Borda rule-assisted collective choice, where members vote the most preferred solution, and (iii) consensual brainstorming deliberation, where the winner of the preceding proposal and voting stages serve as a starting point. The protocol can be used to support modes of democratic management in cultural organisations, by assisting a decision-making practice that enables members to learn their own and others’ preferences while navigating complex, multidimensional choices across organisation strategy, programming and day-to-day operation problems.

## Plain language summary

Cultural organisations and memory institutions – such as museums, cultural centres and community history projects – often face tough choices about how to manage their resources, organise their daily operations and shape their future strategy. These decisions can be complex, involving many voices, perspectives and priorities. Too often, however, decision-making in such organisations remains top-down, leaving staff, contributors and volunteers without a real say. This study presents a new protocol to make decision-making more democratic, inclusive and practical. The approach unfolds in three stages:

(a) Shaping options together – individual preferences are collected and turned into a set of alternative solutions;

(b) Voting with fairness – participants rank alternatives, and the group’s top choices are identified using a voting method known as the Borda rule;

(c) Refining through dialogue – participants deliberate to agree on a final solution that reflects collective consensus.

The protocol is not just theoretical: it was tested in real-world workshops with cultural organisations in Dijon, Athens, and Berlin. The results show that it helps participants make complex decisions in a transparent and collaborative way. By offering a structured yet flexible process, this protocol enables cultural organisations to embrace open, participatory decision-making, supporting and strengthening democratic practice in the sector.

## Introduction

Collective decisions are often assisted, limited and eventually shaped by processes which aim to elicit and finally express individual preferences as much as possible. The obvious challenge in every collective setting is that the final decision will necessarily deviate from each individually desired outcome. This paper, applying a protocol combining a voting and a brainstorming process, argues that individual preferences may evolve during the course of decision-making. In fact, not only verbal exchange and negotiation during a brainstorming session but even the mere observation of others’ ideal endpoints makes the members of a group reconsider their own ideal solutions. This highlights the need for a foundational reconsideration of the way in which collective decision-making has been studied within different strands of the relevant literature in order to account for the dynamics of learning one’s own and others’ preferences in voting and brainstorming processes.

For almost four decades, museum and heritage scholarship, policy and practice have been advocating for more inclusive and participatory cultural and memory institutions, which move beyond top-down management approaches and experiment with democratic models of working with their people and communities. Despite the rich theoretical and empirical groundwork, deep and long-term participation, as praxis embedded in organisational culture and everyday decision-making remains challenging. While participatory work has become present in diverse forms and levels across sectoral practice - such as public consultation, co-creative projects, citizens science - applied examples, where participants wield direct influence over decision-making remain limited ( Mendoza & Talavera, 2025; Strauss, 2022). This enduring gap between participatory ideals and practical implementation highlights a critical need for experimenting with new participatory designs that enable cultural organisations to foster a more open approach to their governance. Along these lines, in this paper we propose a novel protocol for implementing collective decision-making in cultural organisations.

For over two centuries, collective decisions through voting have been extensively studied from theoretical and empirical points of view ( Borda, 1784; Condorcet, 1785; Kurrild-Klitgaard, 2001). Since early contributions, voting theory has been developed through mathematical approaches ( Arrow, 1950; Moulin, 1988) based on anonymity, rationality, perfect information on the alternatives and voters’ strategic competence implying infinite capacity to calculate the consequences of their strategies and those of others. Later, several studies have relaxed some of these simplifications, addressing voters’ bounded rationality, asymmetric information and multi-item agendas ( Jones & McGee, 2024). However, collective decision-making in the real world poses further challenges related to the desire and ability of members to influence the voting agenda and outcome of the decision-making process, voters’ personality or interpersonal relationships issues and asymmetries in the extent to which each member feels engaged in or accountable for the final decision. A plethora of solutions are adopted in collective decision making by committees in private and public institutions all over the world. Often, voting processes are considered too formal or impersonal to be used among peers. We offer an example of how formal voting processes and brainstorming deliberation can be used in a synergetic way, with the former providing a useful starting point for the latter. In fact, introducing an anonymous agenda proposal stage can be the right starting point and a way of mitigating the patronising role of dominant members in consensual brainstorming decision-making sessions.

The decision-making protocol proposed here combines an endogenous project agenda proposal stage, that allows elicitation of individual preferences, followed by a Borda rule-assisted voting for identifying a collectively preferred solution and a group deliberation stage to reach final consensual decision. Our protocol was tested through specially designed workshops with workers and volunteers engaging in cultural and memory work, employed scenarios that allowed us to observe how participants navigate complex, multi-dimensional choices, such as organisational strategies, programmatic directions, and future projects. Our workshops hosted a total of 30 individuals, who contribute to decision-making and management of cultural work in their organisations from three distinct European contexts; Organisation A, which is a contemporary art centre in Dijon, Organisation B, a grassroots network of citizens practicing oral history in Greece, and Organisation C, a LGBTQI+ museum in Berlin.

## The need for new tools and approaches in cultural organisations

Rooted in the legacy of new social movements and the claim for collective action in matters of public interest ( Robertson & Simonsen, 2013), the late 20th century saw the ideal of participation enter the realm of culture and museum work vigorously. Since the rise of ‘new museology’ in the mid-1980s, there have been increased pressures for opening memory institutions to broader publics and their serving as spaces of democratic learning and participation ( Bennett, 2013; Hooper-Greenhill, 1999; Wilson & O’Neill, 2010). Along the same lines, cultural heritage scholarship also critiqued orthodox management approaches as privileging intrinsic, authoritative and Western-centric notions of valuing the past and its resources, thus calling for a participatory turn to accommodate plural and dynamic interpretations in memory work while considering the interests and aspirations of ‘non-experts’ when deciding about the future of cultural heritage ( Chinyele & Lwoga, 2019; Dragouni & Lekakis, 2023; Lekakis & Dragouni, 2020; Smith, 2006; Watson & Waterton, 2010).

In the early 2000s, heritage policy has crystallised the ‘age of participation’ ( Beeksma & De Cesari, 2019), through a series of influential conventions and normative texts, such as the European Landscape Convention ( Council of Europe, 2000), the Universal Declaration on Cultural Diversity ( UNESCO, 2001), the Convention for the Safeguarding of the Intangible Cultural Heritage ( UNESCO, 2003), and the Faro Convention ( Council of Europe, 2005). These soft-law instruments explicitly framed communities as central to the making, valuing and safeguarding of heritage, emphasising community rights, responsibilities and participation as guiding principles for good management practice.

Despite this rich theoretical and policy groundwork, genuine community participation remains elusive and fraught with challenges. While meaningful participatory projects do exist, too often, community involvement is equated with public engagement or awareness-raising, which provides limited opportunities for meaningful debate or authority over strategic decisions ( McQuarrie, 2013; Schiele, 2020; Verdini *et al.*, 2017). Cultural organisations continue to struggle with reaching beyond dominant governance structures and enabling participants to wield genuine influence over decision-making ( Dragouni & Lekakis, 2023), thus risking reducing participation to a tokenistic procedural instrument that legitimises pre-determined decisions ( Beeksma & De Cesari, 2019; May, 2020; Pierroux *et al.*, 2020).

The enduring gap between participatory ideals and practical implementation highlights a critical challenge for both empirical research and professional practice. Long-desired democratic and open participation in organisation and management processes remains difficult to materialise in ways that are equitable and sustainable. As culture and memory organisations in Europe are increasingly expected to increase their resilience, integrate with their surrounding communities and strengthen their democratic profile ( Avdikos *et al.*, 2024), there is a pressing need to experiment with, develop, and evaluate new participatory designs and tools that move beyond rhetoric and enable culture workers and communities to actively co-shape the future of their institutions. This paper contributes to that ongoing discussion, situating itself within the broader trajectory of participatory scholarship while addressing the lack of practical, applicable frameworks for implementation.

## Participatory workshops design and data

### Making decisions for complex, multi-attribute solutions

The workshops employed an experimental technique called “conjoint analysis”, which allows for examining how participants make decisions when faced with multi-dimensional choices ( Franchino & Zucchini, 2015). In our case, such choices relate to future organisation strategies, project-based solutions or new practices, their directions and specifications. Conjoint analysis voting experiments have been used extensively in marketing and economics whereas in the past decade, the technique has been increasingly employed to address questions in political science (see for instance, Christensen, 2020; Hainmueller *et al.*, 2014; Horiuchi *et al.*, 2018; Meyer & Rosenzweig, 2016). In our workshops, conjoint analysis aimed at creating a stimulating environment for individual contemplation and group deliberation allowing participants to express their views and concerns before reaching a consensual decision.

Our protocol deals with several issues emerging in this type of social choice processes. First, in the first stage, the agendas anonymously proposed for voting are protected from personal and strategic effects related to personality issues like leader dominance and identification problems mixing the proposer with the idea. Second, the voting stage is a simple application of voting rules considering a multi-attribute agenda which is endogenously obtained from full membership participation. This is a major innovation of our decision-making protocol, namely creating a starting point for the posterior deliberation among group members that serves as a common ground for a taxonomy of attributes and a unifying vocabulary that assist the deliberation process. The latter can be compatible with and even assisted by the rational social choice process preceding it.

### From real problems to realistic scenarios

Before designing the scenarios of our workshops, we reached out to our liaisons with participants/organisations, discussing extensively with them about their current organisational dynamics and challenges. This helped us to design the workshops in a way that was both meaningful for our research and relevant to our participants, aligning work scenarios with realistic programmatic directions and/or actual projects that participants sought to materialize in their teams.

***Case/Workshop #1: Organisation A, Dijon.*** Organisation A (henceforth, OrgA) is a contemporary art centre and museum in Dijon, established in 1977. OrgA operates as an independent non-profit organisation and is sustained by a small team of paid staff. Apart from promoting avant-garde contemporary artwork, OrgA is missioned to support young artists and artistic freedom through its curatorial practice. OrgA acts as producer and/or intermediary for many local creators while supporting co-curatorial practice and public engagement. However, as museum visitation numbers were relatively low, at the time of our research, OrgA’s management sought alternative solutions for using resources and spaces efficiently, to increase its impact on artistic creativity and audiences. Along these lines, we devised a work scenario that concerned future development options for OrgA including its funding strategy, collections’ handling and its allocation of facilities for new uses.

***Case/Workshop #2: Organisation B, Greece.*** Organisation B (henceforth, OrgB) is a grassroots network of citizens and non-professional historians, formed in 2011. Although originally set in Athens, the OrgB community has grown steadily in geographic scope, hosting today more than 20 active teams from across Greece. OrgB operates as an autonomous collective that is sustained by the volunteer contributions of its members, who collect life stories, testimonials and other oral history data, organised and stored in distinct (team) records. At the time of our research, the network was exploring solutions for data sharing and/or related infrastructure in order to secure the archives’ sustainability, openness and accessibility to a broad audience. Based on this, we created a work scenario that proposed the development of a digital platform, exploring options regarding its capacities and features, as well as its implementation and maintenance strategy.

***Case/Workshop #3: Organisation C, Berlin.*** Founded in 1984 by activist communities, Berlin’s Organisation C (henceforth, OrgC) has been safeguarding and promoting the histories and memories of LGBTQI+ communities, through its archival work, exhibitions and public events. Run through a non-profit ‘Friends of the Museum’ association, OrgC’s organisational model blends volunteer leadership with professional practice. Daily operations, curatorial work and research activities are performed collaboratively by its permanent staff and its multitudinous community of volunteers, comprising a mix of senior members and newcomers. At the time of our research, OrgC wished to upgrade some of the museum’s spaces and amenities while re-organising volunteer work to secure smooth operation and address demands for greater transparency and participation. Having this in mind, a work scenario was developed to reflect shift staffing policy and options regarding volunteers’ allocation rules, teams’ composition and priority rules (e.g., based on loyalty).

### Protocol

All workshops followed the same protocol, inviting participants to perform the same decision-making tasks, as framed by a tailored work scenario, which, as we described earlier, reflected their actual needs and agendas. Following a short introduction – description that welcomed participants and provided them with some basic instructions, the session was organised in three tasks:

***Task A: Submitting a candidate multi-attribute solution.*** In this task, participants were provided with a table-menu with different factors, framing the future solution under consideration. Under each factor there were some pre-set options and a blank field marked as ‘other option’. Participants were asked to work individually (and silently) with the view to build their ideal proposal (anonymously), by choosing their most preferred option under each factor. They could freely choose one of the pre-set options or propose one of their own.

After completing the task, the researchers collected the responses, assigned them with a code and moved on to create a tableau where all proposals could be seen by all participants.

***Task B: Ranking of proposed solutions through voting.*** In the second individual task, all participants were allowed a few minutes to review all the anonymous proposals. Each participant was then requested to anonymously rank all proposals from their most to their least preferred, by assigning 12 points to their first choice, 10 points to their second choice, 8 points to their third choice and 7, 6, 5, 4, 3, 2, 1 points to the rest of the proposals according to their judgement. This is a version of the Borda rule.

After the completion of Task B, the researchers collected all votes and calculated the results; all results were then exhibit publicly, and the winner proposal (most voted) was presented to participants.

***Task C: Deliberation and refining of solution.*** In the final task, participants were invited to review the winner solution along with the list of proposals as ranked by voting and deliberate freely on the components and specifications that emerged during the process. They were free to keep the winner solution unchanged or modify any (or all) of its features to fit with their collective preferences. Brainstorming and deliberation were proposed for an approximate time horizon of 30 to 60 minutes but without any strict rule enforcing this timing. Rather, participants were allowed to choose freely the duration and moderation rules of their discussion. The researchers did not participate or otherwise intervene in the task, unless for providing some clarification of the procedure, if and when requested. The discussion was recorded anonymously to an audio file. At the end of the session, participants submitted their final solution - outcome.

### Implementation and data

The workshops were held between April and October 2024 in Dijon, Athens and Berlin, respectively. Our pool of participants was limited to individuals contributing directly to the work of the organisations under consideration, having an active role in the management, daily operation and/or production and delivery of related cultural and memory work. A total of 30 individuals contributed to the workshops.

The workshops produced three sets of data (one for each case), both quantitative and qualitative. Quantitative data provided information about participants’ individual preferences towards the topics discussed. Along with voting scores, data on preferences show how participants form their preferences and make decisions about alternatives ( Hainmueller *et al.*, 2014). To deepen our understanding of this process, we also recorded group brainstorming and deliberation. These qualitative data were transcribed and used to shed further light into the process of refining and re-building the “winner” solution into a final proposal - outcome, by common consensus. All data were anonymous and each participant was assigned a code.

## Results

The Dijon workshop produced eight (8) alternative combinations (proposed solutions) in Task A, reflecting participants’ distinct preferences regarding funding strategies, collections management and the future development of OrgA. During voting (Task B), two of these solutions tied as the most collectively preferred (winner proposals), each receiving 66 points, while the least-preferred proposal obtained 33 points. Interestingly, the two winner proposals diverged in their approach to collections management and reuse of spaces. The first advocated transferring assets to other institutions or even private collectors, thereby freeing space for artist residencies and for on-site exhibitions showcasing contemporary artwork. The second favoured retaining, digitising and archiving the collections, while envisioning OrgA’s transformation into a learning space in partnership with a School of Art.

The final outcome adopted after deliberation (Task C) represented a compromise between these two proposals: it excluded asset transfers but endorsed the hosting of on-site exhibitions. These results suggest that participants’ initial preferences often shift following peer discussion, and that voting outcomes may not necessarily capture either individuals’ ideal choices or post-deliberation collective solutions.

The Athens workshop produced eleven (11) alternative solutions in Task A, corresponding to the number of participants. In Task B, the most-voted proposal received 71 points (as compared to the second most-voted that received 67 points and the least-voted that received 42 points). The winner proposal obtained both the highest total score and the highest number of top rankings (12 points). A comparison between individual preferences (Task A) with voting data (Task B) indicates that the most-voted solution largely reflected majority views on digital platform design, media and user interaction. Exemptions emerged in relation to data rights, where we observed some disaccord around open-data policy. Minor divergences also appeared with regards to project implementation and maintenance: while there was wide consensus that OrgB should implement the project with external support, opinions differed on the profile of the ideal partner (e.g., public or private institution).

After deliberation (Task C), however, participants revised all platform features and broadened the range of preferred implementation options. Interestingly, data from group discussion and debate revealed that some earlier choices had been made without a full understanding of the concepts involved. For example, participants held differing interpretations of what constitutes a ‘digital museum’. Deliberation helped establish shared meanings and align strategy with collective priorities. As transcripts indicate, participants expressed a strong desire to balance curated experiences with user autonomy, leading them to propose a ‘hybrid’ strategy that would offer multiple layers of organising materials on the platform. Another fascinating example concerned user capacities and data rights: while the most-voted proposal promoted a more interactive communication and commenting for the virtual visitor, the ethical concerns which emerged during group deliberation led the final proposal to limit user participation. Similarly, although Creative Commons non-commercial licensing initially received broad support, deliberation exposed misconceptions regarding about open-data tools and conflicting views within OrgB regarding data ownership.

The Berlin workshop produced ten (10) distinct solutions in Task A, capturing participants’ preferred options for reorganising volunteer work and staffing arrangements at OrgC. A majority favoured fixed task allocation (50%), self-entered shifts (60%) and teams of 4–5 members (60%). Furthermore, 70% prioritised senior volunteers over newcomers. These preferences were reflected in the most-voted proposal (Task B), which received 64 points. Regarding the composition of volunteer groups, the majority (40%) chose fluid and homogenous teams in Task A, whereas the exact opposite (fixed and diverse teams) was recommended in the most-voted winner proposal (Task B). Notably, the winner proposal polarised participants: it received very high scores by six voters (12-10-10-10-10-8 points), but was rated poorly by the remaining three (1-1-2 points).

It is therefore not surprising that the final outcome after deliberation (Task C) diverged substantially from the winner proposal (Task B). Group discussion prompted significant shifts across all dimensions considered. Although fixed tasks were acknowledged as practical, participants emphasised the need for flexibility and autonomy in task crafting. Preferences for self-entry allocation shifted toward allocation by request. Collective reflection revealed that maintaining the existing system was a reasonable option, as it was perceived to be functioning effectively. Debate also reframed the meaning to “diversity”: rather than focusing on team composition, participants emphasised the value of intergenerational learning. Mixing senior and new members was viewed as an opportunity for the latter to integrate into the organisation. Seniority-based prioritisation was ultimately rejected; instead, reliability was identified as the fairest criterion for allocation. Finally, preferences shifted toward smaller teams, as participants highlighted that trust and a sense of security are grounded in close personal relationships.

An overview of descriptive statistics for each workshop/case is provided in Table 1. As shown by these statistics, participants’ preferences did not remain static during the process. For example, we see that in Dijon half of the participants (0.5) did not cast most votes on their own proposal but rather chose an alternative solution ( *max votes to own proposal*). In Athens and Berlin, a shift of preferences is also observed for several participants. In addition, during the decision-making in Athens and Berlin workshops, the “distance” between individual preferences and collective choice has increased from voting (winner project) to brainstorming deliberation (final outcome) and consensual decision. This indicates that flexibility allowed fermentation and learning during the process, as participants were able to navigate through other alternatives and negotiate options without being bound to the “tyranny” of their initial individual preferences. This fertile process did not prevent the groups from achieving their main objective, which was reaching consensus over their future projects and directions.

**Table 1.** Aggregate descriptive statistics on proposal submission, voting outcomes and brainstorming sessions.

	Dijon *	Athens	Berlin
Winner project = Final outcome	No (No)	No	No
If No, % difference **	0.3 (0.2)	0.48	0.5
Max votes to own proposal	0.50	0.82	0.7
Distance (median) of winner project ***	4.0 (2.0)	8.0	4.0
Distance (median) of final outcome	1.0	9.0	8.0

*Notes:* * Dijon produced two winner projects in the voting task (Task B). Results for winner scenario 2 are reported in brackets. **Based on the number of traits featuring in the work scenario. ***Distances from individual choices, as made in Task A.

## Discussion and concluding remarks

This paper presents results on collective decisions-making in three different cultural organisations. The results of the workshops provide valuable insights into the internal processes of participatory decision-making in culture and memory work, whereas the observed patterns may also apply to other similar contexts. Above all, the observed dynamics during the voting and the consensual brainstorming sessions suggest that our decision-making protocol serves as a fermentation mechanism for individual and collective preferences, rather than a static platform for the transformation of unambiguous individual preferences into collective decisions.

First, our research focused on a small peripheral museum (OrgA) that is challenged by limited visitation and low public engagement with its permanent collections. OrgA has also relatively low experience in participatory governance, as managerial decisions follow a horizontal hierarchy (co-directors). Nevertheless, we observed that opening the discussion of critical matters to the broader team proved beneficial and inspiring, since staff members contributed creative solutions for the organisation’s future, firmly grounded in its mission and ethos.

At the time of the study, OrgA was exploring strategies to strengthen its impact on surrounding communities, while remaining true to its commitment to supporting young artists and artistic freedom through its curatorial practice. Our data suggest that both co-directors and staff agree on departing from traditional museum practices and experimenting with new uses of space and resources to better support artistic creativity, provide training and promote emerging artists. However, these ideas are still fluid and not yet consolidated, requiring further elaboration and development of the specifications for a new multi-layered strategy that will address challenges which are common among small peripheral cultural and memory institutions. Accordingly, the protocol applied here may serve as an entry point for initiating dialogue in cultural organisations that have limited experience in participatory processes.

Second, our work examined a grassroots oral history network (OrgB) with considerable familiarity in participatory governance and collective decision-making. OrgB operates through vertical non-hierarchical structures and commoning practices (e.g., voluntary contributions, porous community). Discussion here centred on the design of a digital platform, deemed critical for long-term sustainability and data accessibility. Issues of sharing and openness represent persistent challenges for oral-history projects, especially when operating autonomously without institutional support. The participatory decision-making protocol applied in the workshop revealed uneven levels of understanding among members in areas extending beyond the core of cultural/memory work – such as digital literacy, intellectual property rights and OpenGLAM principles. Considering the lack of shared knowledge, conflictual arguments and debate on access and control of materials, deliberation was essential for establishing a common understanding and generating creative combinatory solution that better serve the collective purpose – for example, the multi-layered organisation of materials to accommodate different needs and uses.

Third, we examined a case positioned between OrgA and OrgB on the participatory spectrum. OrgC has a long tradition of volunteer work where decisions are formally made by a board of directors, yet with substantial input and representation from the community. Dialogue with volunteers proved highly effective for co-designing organisational arrangements directly impacting participants, specifically volunteer labour and shift staffing, which are critical for the museum’s daily functioning. As demands for greater transparency and participation grow, OrgC’s internal processes need to be adapted to enable more flexible structures and mechanisms for internal learning and communication. Interestingly, deliberation among participants regarding task distribution, volunteer roles and team composition surfaced existing dynamics between longstanding and newer volunteers. The protocol applied here helped address disparities in power between established and newer members, particularly in response to younger volunteers’ calls for more frequent role rotation and equal voice. Moreover, deliberation allowed critical issues to be articulated, including the need for task crafting and relational crafting, where participants have the freedom to proactively shape both their roles and social interactions within the museum.

Overall, the transition from the elicitation of individual preferences to voting confirmed rationality at an individual level, as most (but not all) participants assigned maximum points to their own proposal. At the same time, the finding that some voters did not vote their own proposal as the best after alternative proposals were publicly revealed suggests a clear violation of classical notions of rationality. But in a broader framework of rationally learning agents, this finding may simply imply that under bounded rationality a voter may have failed to consider some issues which are reflected on another group member’s ideal endpoint. One could assume that this may simply be a pattern which is specific to some kind of preference ambiguity, regret or even regression to the mean. Then, collective decisions which are based on the pursuit of consensus through brainstorming should bring the final decision closer to the group’s initial preferences as aggregated from initial proposals. This is not the case, according to our results.

Deliberation sessions consistently led to consensual choices that differed from both initial proposals and the voting outcome in each of the cases studied. In fact, there is mixed evidence, with some brainstorming sessions reaching decisions deviating less from initially elicited preferences (Dijon), but in other cases there is no significant convergence (Athens) or even a larger convergence as compared with the voting session’s “winner” solution (Berlin). Despite this divergence, consensus-building remained the preferred route, and both processes (voting and deliberation) led to final outcome, irrespective of the distance from the initially declared individually preferred endpoints. Importantly, the individual proposal and voting stages created a common language and a productive starting point, thereby facilitating the consensual final choice through the brainstorming session.

The approach outlined in this paper constitutes a methodological proposal for the management of decision-making processes in the framework of more participatory governance of cultural organisations. The method proposed here consists of three stages: an endogenous project agenda proposal (individual preferences), followed by Borda rule-assisted voting (collective selection) and finally group deliberation. Our decision-making protocol introduces several key innovations. First, during the agenda-setting phase, proposals are anonymised to minimise biases associated with leader dominance or the identification of ideas with specific participants. Second, we employed a rigorous voting mechanism that integrated diverse attributes into the agenda, obtained endogenously from participants’ input. This design encourages deep deliberation, grounded in a shared understanding of attributes and a unifying vocabulary for democratic discussion, ultimately facilitating consensus.

The initial stage guarantees that preservation of individual proposals into the voting stage, shielded from strategic manipulation through simultaneous submission and proposers’ anonymity. This design addresses the familiar pathology of representative electoral systems, where dominant parties prioritise identity and loyalty stronger over content, marginalising weaker voices and suppressing potentially strong ideas. The voting stage confirmed the rationality of individual proposers: The vast majority ranked their own proposals first.

The application of the Borda Rule, as in many contexts, was sufficient to identify the collectively preferred solution. Nevertheless, this ‘final’ choice-by-voting did not remain intact during deliberation. In all cases, group discussion through real-time interaction and face-to-face verbal exchange yielded unanimously accepted solutions that voting alone could not achieve. The voting stage was not unnecessary; rather, it provided the foundation for individual participation, a democratically structured set of ideas, and a vocabulary of focal points for subsequent deliberation.

Our method thus combines traditional elements of democratic decision-making with novel aspects that are both logistically feasible and socially efficient. Further research is needed to validate these preliminary findings across a broader variety of contexts and larger groups. Extending this approach to larger scales and applying it systematically to internal organisation structures will require more technologically intensive infrastructure than the one employed in the workshops reported here.

The protocol examined here has practical relevance for cultural and memory organisations that wish to embed collective decision-making beyond ad hoc consultation. Its sequence – anonymous agenda-setting, Borda-assisted ranking, and subsequent deliberation – appears sufficiently flexible to be adapted across different sizes, governance models and resource levels.

In terms of readiness and enabling conditions for the protocol’s implementation, successful implementation is more likely where the choice architecture is co-designed with prospective participants, for example through brief piloting of attributes and definitions to secure common understandings. Fairness safeguards (such as anonymity during the proposal stage and rotating facilitation during discussion) tend to reduce status effects and idea–owner identification. The protocol seems especially pertinent for multi-attribute decisions with cross-team consequences (e.g., space use, digital platform parameters, or volunteer rostering), programme development and prioritisation, and operational policies that benefit from local knowledge and organisational buy-in. For larger organisations, a federated arrangement may be suitable: units undertake local rounds and forward their strongest proposal(s) for a cross-unit refinement stage. Low-tech solutions (forms plus spreadsheet tallies) generally suffice, although dedicated tools can support anonymity and version control. Asynchronous windows for submission and review could broaden reach without increasing meeting load.

Across the workshops reported here, final consensual outcomes frequently diverged from both initial proposals and the ranked leader. Rather than signalling aggregation failure, this pattern may be interpreted as preference learning: private proposal stages reduce conformity pressures; exposure to structured alternatives expands the option space; and discussion clarifies meanings and aligns choices with shared values. Recording reasons and trade-offs provides a defensible basis for organisational legitimacy.

Attention to internal dynamics appears important. Rotating (co-)facilitation, speaking-order tools, explicit invitations to first-time or junior contributors, and transparent criteria for how proposals advance may mitigate dominance effects. Where long-standing and newer members coexist, paired roles or mentoring structures could institutionalise intergenerational learning and ease integration.

To avoid one-off exercises, organisations might establish a cadence (e.g., periodic cycles aligned to programming), maintain a small pool of trained facilitators, and keep a living repository of decisions and attribute menus that lowers future transaction costs. The protocol is best viewed as complementary to statutory governance and expert judgement. Clarity about decision rights at the outset—what is binding, what is advisory, and what constraints apply—appears essential so that participation enhances, rather than obscures, accountability.

Overall, the evidence suggests that relatively modest process innovations can support the articulation of collective choices which are inclusive and legitimate, while also building shared vocabularies and routines that accumulate value over time. While challenges remain, the evidence presented here demonstrates that even small-scale, carefully designed interventions can foster meaningful change. Ultimately, the findings from these workshops highlight the transformative potential of structured participatory processes in cultural organisations. By balancing individual expression with collective deliberation and flexibility, such methods can unlock creativity, strengthen organisational cohesion, and support more democratic governance. In this sense, participatory decision-making does not merely address immediate organisational needs but also nurtures cultures of collaboration and trust that are essential to the resilience of cultural and memory institutions.

Future research should pay more attention to the evolution and learning on and from one’s own and others’ preferences over negotiation and decision-making processes. After all, collective decisions are not, cannot and should not be seen as a static mapping from individual starting endpoints to a final decision, but rather as a dynamic process of learning about one’s own, others’ and eventually, collective preferences as they evolve and ferment in a constructive golden braid.

## Ethics and consent

All the empirical research with human subjects has gained ethics approval by the Ethics Committees of participating universities. The field experiment analysed in this article was approved by the Ethics Committee of Panteion University of Social and Political Sciences (approval number 30/7-6-2022) and by the Burgundy School of Business Ethics Committee (approval numbers CERBSB2023-55 and CERBSB2023-63). The field experiment did not involve deception. Participants were provided with both verbal and written explanations of the field experiment and an informed consent form. Participation was voluntary, and participants were informed about their right to withdraw from the study at any time without providing an explanation and without any repercussions. Researchers followed data protection and confidentiality protocols. Research data were anonymous. Participants in the field experiment were all adult and provided informed consent in writing before their participation.

## Data Availability

SoDaNet Data Catalogue: A participatory protocol for collective decision-making in cultural organisations. https://doi.org/10.17903/FK2/NHLCH9 The project contains the following underlying data: Codebook.pdf (Description of variables and codes used in the dataset) CSV_Dataset_Cases_1_3.rar (Dataset of the 3 cases in CSV format) EXCEL_Dataset_Cases_1_3.rar (Dataset of the 3 cases in Excel format) No extended data are associated with this article.
