# Supplementary material for: A participatory protocol for collective decision-making in cultural organisations
Source: Open Res Eur. 2026 Jul 15;5:339. Originally published 2025 Nov 6. [Version 2] doi: 10.12688/openreseurope.21601.2 (PMC13555065; doi:10.12688/openreseurope.21601.2)
Supplement: Peer review history

# Reviewer report: Reviewer response for version 2

**DOI:** 10.21956/openreseurope.26511.r77441

**Published:** 2026-07-23

**Authors:** Deniz Ikiz Kaya

**Affiliations:** 1 Eindhoven University of Technology, Eindhoven, The Netherlands

I have reviewed the revisions made and the response letter, and I agree with all the changes.

 Hence, I agree to changing its status to approval. I don’t have any further comments.

If any, are all the source data and materials underlying the results available?

Yes

Is the work original in terms of material and argument?

Yes

Is the work clearly and cogently presented?

Yes

Is the argument persuasive and supported by evidence?

Yes

Does the research article contribute to the cultural, historical, social understanding of the field?

Yes

Does it sufficiently engage with relevant methodologies and secondary literature on the topic?

Yes

Reviewer Expertise:

NA

I confirm that I have read this submission and believe that I have an appropriate level of expertise to confirm that it is of an acceptable scientific standard.

# Reviewer report: Reviewer response for version 1

**DOI:** 10.21956/openreseurope.23369.r64491

**Published:** 2025-12-19

**Authors:** Niki Nikonanou

**Affiliations:** 1 University of Thessaly, Thessaly, Greece

The research article presents a compelling and well-documented study. It proposes a structured protocol for participatory decision-making in cultural organizations and reports on empirical research examining its implementation in three European cultural organizations. The methodology is innovative and original, demonstrating satisfactory effectiveness and offering a tangible alternative for participatory decision-making—at least within certain operational domains of cultural organizations—while contributing to their democratization and sustainability. Particularly noteworthy is the application of the protocol in real work senarios, addressing actual organizational challenges and projects during the implementation period. However, it would be valuable to explore the inclusion of ‘non-expert’ participants in these procedures to further enhance their inclusivity. Furthermore, a more in-depth analysis of the research findings concerning how the protocol addressed inequalities and power relations within the participating groups would strengthen the study.

 The literature review engages with relevant research addressing the challenges faced by cultural organizations in their efforts toward democratization through participatory processes, as well as the ambiguities and limitations often associated with the broad use of the term “participation.” However, a stronger engagement with theory of the commons would be beneficial, particularly in clarifying whether and how it informed the design of the proposed protocol. In addition, a more in-depth analysis of the organizational structures of the participating institutions would strengthen the study. Although some aspects of  governance and operation  of the institutions are mentioned, these features are not sufficiently examined in relation to how prior experience may have influenced the implementation of the protocol.

 The article is clearly and coherently presented, and its argument is persuasive. Overall, the study makes a valuable contribution to the field, and I recommend its indexing with minor revisions.

If any, are all the source data and materials underlying the results available?

Yes

Is the work original in terms of material and argument?

Yes

Is the work clearly and cogently presented?

Yes

Is the argument persuasive and supported by evidence?

Yes

Does the research article contribute to the cultural, historical, social understanding of the field?

Yes

Does it sufficiently engage with relevant methodologies and secondary literature on the topic?

Partly

Reviewer Expertise:

theory and practice of museum-education,  social dimension of museums and cultural institutions, participation and inclusion in culture, educational and cultural commons

I confirm that I have read this submission and believe that I have an appropriate level of expertise to confirm that it is of an acceptable scientific standard.

# Response

**Published:** 2026-06-30

**Authors:** Mina Dragouni

**Affiliations:** Department of Communication, Media & Culture, Panteion University of Social and Political Sciences, Athens, Attica, Greece

Dear Prof Nikonanou, We sincerely thank you for your time and for the thoughtful feedback you have provided. We have given thorough consideration to each of your remarks and have amended our manuscript accordingly. In the following lines, we provide our detailed responses to your observations. Again, thank you very much for reviewing our paper!    

***Reviewer’s comment:*** *The research article presents a compelling and well-documented study. It proposes a structured protocol for participatory decision-making in cultural organizations and reports on empirical research examining its implementation in three European cultural organizations. The methodology is innovative and original, demonstrating satisfactory effectiveness and offering a tangible alternative for participatory decision-making—at least within certain operational domains of cultural organizations—while contributing to their democratization and sustainability.* *Particularly noteworthy is the application of the protocol in real work senarios, addressing actual organizational challenges and projects during the implementation period. However, it would be valuable to explore the inclusion of ‘non-expert’ participants in these procedures to further enhance their inclusivity.*

**Authors’ response: **Thank you very much for this observation. Please note that the workshops held in Athens and Berlin engaged ‘non-expert’ participants who serve as volunteers rather than professional cultural workers. We have updated the introduction to illustrate this more clearly – “We test our protocol through specially designed workshops with cultural workers and volunteers, engaging a total of 30 expert and ‘non-expert’ participants from three distinct cases in Europe”.

***Reviewer’s comment:*** *Furthermore, a more in-depth analysis of the research findings concerning how the protocol addressed inequalities and power relations within the participating groups would strengthen the study.*

**Authors’ response: **Thank you very much for this crucial point. Power inequalities were primarily addressed through the anonymous agenda-setting stage (Stage 1 of the protocol). As detailed in the text “in the first stage, the agendas anonymously proposed for voting are protected from personal and strategic effects related to personality issues like leader dominance and identification problems mixing the proposer with the idea.” Indeed, our findings verify the “preservation of individual proposals into the voting stage, shielded from strategic manipulation through simultaneous submission and proposers’ anonymity.” The mitigation of pre-existing power imbalances was directly observed as the workshop tasks unfolded and was subsequently corroborated by our results. For example, regarding the Berlin workshop, we note: “deliberation among participants regarding task distribution, volunteer roles and team composition surfaced existing dynamics between longstanding and newer volunteers. The protocol applied here helped address disparities in power between established and newer members, particularly in response to younger volunteers’ calls for more frequent role rotation and equal voice.” (Discussion, para 5) – This is directly reflected in the outcomes produced by the participants: “rather than focusing on team composition, participants emphasised the value of intergenerational learning. Mixing senior and new members was viewed as an opportunity for the latter to integrate into the organisation. Moreover, seniority-based prioritisation was ultimately rejected; instead, reliability was identified as the fairest criterion for allocation.” (Results, para. 6)

***Reviewer’s comment:*** *The literature review engages with relevant research addressing the challenges faced by cultural organizations in their efforts toward democratization through participatory processes, as well as the ambiguities and limitations often associated with the broad use of the term “participation.” However, a stronger engagement with theory of the commons would be beneficial, particularly in clarifying whether and how it informed the design of the proposed protocol.*

**Authors’ response: **We appreciate this thoughtful suggestion; however, we have chosen to not engage with the theory of the commons in this paper. Because commons theory did not directly inform the design of the proposed protocol, introducing it would expand the scope and risk diluting our core arguments.

***Reviewer’s comment:*** *In addition, a more in-depth analysis of the organizational structures of the participating institutions would strengthen the study. Although some aspects of  governance and operation  of the institutions are mentioned, these features are not sufficiently examined in relation to how prior experience may have influenced the implementation of the protocol.*

**Authors’ response: **We agree that this context is valuable. Accordingly, we have added further details regarding the organisations’ prior experience with participatory governance in the opening paragraph of the sub-section ‘ *From real problems to realistic scenarios’*.

# Reviewer report: Reviewer response for version 1

**DOI:** 10.21956/openreseurope.23369.r63787

**Published:** 2025-12-08

**Authors:** Deniz Ikiz Kaya

**Affiliations:** 1 Eindhoven University of Technology, Eindhoven, The Netherlands

Dear Authors,

 Thank you for submitting your research article, it has been an engaging read.

 This paper addresses an important gap in the governance and operation of cultural organisations—specifically, the lack of practical, transparent, and democratic tools that embed participation in everyday decision-making. The authors propose and experimentally test a participatory decision-making protocol combining anonymous agenda formation, Borda rule–based voting, and consensus-oriented deliberation. The topic is timely, as participation is gaining greater significance across the sector, and the contribution is potentially valuable for cultural institutions striving to operationalise inclusive governance.

 Please find below my extended comments and suggestions for improvement for each section of your manuscript.

 I wish you good luck with the revisions.

**Keywords:**

- Some keywords are synonymous, such as participation and participatory decision-making. I would also suggest being more specific regarding the participatory tools, such as consensus building or Borda voting.

**Introduction: **

- The introduction begins by discussing the evolution of individual preferences during collective decision-making. This is an interesting premise, but the link between this general theoretical claim and the specific domain of cultural organisations becomes clear only later. Perhaps, you can strengthen the framing by more explicitly signalling early on that the paper contributes to participatory governance in cultural and heritage organisations. This will help readers anticipate the relevance and context of the argument from the beginning.
- In the first two paragraphs, there are strong theoretical claims, such as the limitation of inclusive governance practices. While these arguments are compelling, they currently lack empirical grounding, especially from within museum and heritage studies, where participation, organisational change, and inclusive governance are extensively debated. I would suggest incorporating more references to key sources to reinforce the credibility of these arguments early on.
-  The theoretical transitions can be smoother. For example, the text moves from voting theory and preference formation to participatory governance in museums, and then to formal voting theory. You can consider adding bridging sentences and explicitly state that the paper seeks to bridge two bodies of literature: social choice/voting theory and participatory cultural governance.
- It is stated that the paper offers a “novel protocol” but could be clearer about what is new compared to existing participatory methods and why.

**The need for new tools and approaches:**

- Overall, this section reads more as a chronological literature review on the theory and policy history of participation within cultural and heritage sectors. However, the argument would benefit from being more explicitly synthesized, and it would be better to draft it as an analytical build-up toward the core argument of the paper.
- The section provides a solid grounding in participatory governance debates, but there is still a conceptual gap between the first part of the introduction (on evolving preferences, voting, and governance theory) and this literature-driven overview of participation in cultural organisations. Please clarify how the argument connects to the earlier discussion on preference formation.
- The section argues convincingly that genuine participation remains limited, often devolving into engagement without influence. However, some statements could be strengthened with clearer empirical grounding.
- The section leans heavily on policy instruments, but not much on the organisational practice literature. You can draw on literature that examines internal organisational dynamics and decision making. This would strengthen the bridge between policy aspirations and everyday organisational realities, reinforcing the relevance of a decision-making tool designed for internal use. 

**Participatory workshops design and data:**

 Overall, this section reads primarily as the methodological description of the study, outlining the organisational context (OrgC), the workshop protocol, and the data collection process. It is clear and detailed; however, several aspects could be strengthened to deepen its analytical depth and better connect it to the broader research aims.

- Clarify methodological positioning: The section could more explicitly signal how these workshops function within the overall research design. Currently, the narrative is descriptive, but the methodological rationale could be foregrounded more clearly.
- Strengthen the link between organisational context and analytical framing: The description of OrgC is informative, but it could connect more explicitly to why this organisational setting matters methodologically. Briefly connecting these characteristics to decision-making challenges would better justify the choice of case.
- Show how the tasks connect to organisational inclusion: The workshop protocol is well-structured, but the feedback would be stronger if you could briefly explain why the three-task sequence was chosen, how it captures or disrupts existing organisational power patterns, how it supports more inclusive governance routines (especially in heritage settings where community voices are often marginalized etc).
- Describe how the qualitative data is analyzed in details.

**Results:**

 Overall, the results are clearly articulated and provide compelling evidence. However, the section would be significantly strengthened by making more explicit how the three-task structure contributes to organisational inclusion and participatory governance.

- The results describe what happened in each workshop, but the manuscript would benefit from a clearer articulation of why Task A (individual proposals), Task B (voting), and Task C (deliberation) matter for inclusive governance. (how do they relate to each other)
- The results illustrate cumulative learning and movement toward consensus, but the implications for ongoing governance could be made more explicit.

**Discussion and conclusion:**

 Overall, this section is empirically robust, synthesizes findings across the three cases, and clearly highlights both the methodological contribution of the decision-making protocol and its practical implications for cultural and heritage organisations.

 To strengthen the section, you may consider clarifying the through-line that connects your empirical findings to broader theoretical claims about participatory governance, organisational inclusion, and preference formation (referring back to the introduction and the literature review).

 Some arguments would benefit from being brought into sharper dialogue with the heritage governance literature, and certain interpretive claims may need tighter framing or more explicit justification. For example, the metaphor of “fermentation” is compelling but may require more conceptual scaffolding.

If any, are all the source data and materials underlying the results available?

Yes

Is the work original in terms of material and argument?

Yes

Is the work clearly and cogently presented?

Yes

Is the argument persuasive and supported by evidence?

Yes

Does the research article contribute to the cultural, historical, social understanding of the field?

Yes

Does it sufficiently engage with relevant methodologies and secondary literature on the topic?

Yes

Reviewer Expertise:

heritage governance, participatory tools and decision-making

I confirm that I have read this submission and believe that I have an appropriate level of expertise to confirm that it is of an acceptable scientific standard, however I have significant reservations, as outlined above.

# Response

**Published:** 2026-06-30

**Authors:** Mina Dragouni

**Affiliations:** Department of Communication, Media & Culture, Panteion University of Social and Political Sciences, Athens, Attica, Greece

Dear Dr Deniz Ikiz Kaya, We really appreciate your time to review our paper and we thank you very much for your insightful feedback. We have carefully considered all your comments and have amended our article accordingly. In the following lines, we provide a detailed response outlining the changes made in response to each of your observations. We hope that the revisions adequately address your comments and once again, we thank you very much for reviewing our article!    

**Reviewer’s comment:** *Some keywords are synonymous, such as participation and participatory decision-making. I would also suggest being more specific regarding the participatory tools, such as consensus building or Borda voting.*

**Authors’ response:** Thank you very much for this helpful observation. We have revised our keywords accordingly.

**Reviewer’s comment:** *The introduction begins by discussing the evolution of individual preferences during collective decision-making. This is an interesting premise, but the link between this general theoretical claim and the specific domain of cultural organisations becomes clear only later. Perhaps, you can strengthen the framing by more explicitly signalling early on that the paper contributes to participatory governance in cultural and heritage organisations. This will help readers anticipate the relevance and context of the argument from the beginning.*

**Authors’ response:** We agree that the framing in the original manuscript required greater clarity. To address this, we have thoroughly revised the introduction to better position our narrative within the literature on participatory governance in cultural organisations.

**Reviewer’s comment:** *In the first two paragraphs, there are strong theoretical claims, such as the limitation of inclusive governance practices. While these arguments are compelling, they currently lack empirical grounding, especially from within museum and heritage studies, where participation, organisational change, and inclusive governance are extensively debated. I would suggest incorporating more references to key sources to reinforce the credibility of these arguments early on.*

**Authors’ response:** Following your recommendation, we have revised the first 2 paragraphs of the introduction and integrated additional references from Museum and Heritage Studies. A more detailed review of this literature is provided in the subsequent section of the article, titled: “The need for new tools and approaches in cultural organisations”.

**Reviewer’s comment:** *The theoretical transitions can be smoother. For example, the text moves from voting theory and preference formation to participatory governance in museums, and then to formal voting theory. You can consider adding bridging sentences and explicitly state that the paper seeks to bridge two bodies of literature: social choice/voting theory and participatory cultural governance.*

**Authors’ response:** We have restructured and enriched the introduction to improve its overall clarity and flow. We believe these revisions address prior transition issues.

**Reviewer’s comment:** *It is stated that the paper offers a “novel protocol” but could be clearer about what is new compared to existing participatory methods and why.*

**Authors’ response:** To address this, we have added a new paragraph (par 5) to the introduction (beginning with “In particular, our decision-making protocol combines…”), to explicitly highlight the novel aspects of the protocol. This addition also clarifies the specific dynamics we intended to explore during the workshops.

**Reviewer’s comment:** *The need for new tools and approaches* *-* *Overall, this section reads more as a chronological literature review on the theory and policy history of participation within cultural and heritage sectors. However, the argument would benefit from being more explicitly synthesized, and it would be better to draft it as an analytical build-up toward the core argument of the paper.*

**Authors’ response:** We appreciate this insightful comment; however, our intention was to synthesize this content specifically to illustrate the enduring gap between participatory theory and its materialization in sectoral practice. Our underlying argument is that new decision-making designs can enrich the participatory toolbox and help addressing this gap.

**Reviewer’s comment:** *The section provides a solid grounding in participatory governance debates, but there is still a conceptual gap between the first part of the introduction (on evolving preferences, voting, and governance theory) and this literature-driven overview of participation in cultural organisations. Please clarify how the argument connects to the earlier discussion on preference formation.*

**Authors’ response:** Thank you very much for bringing this to our attention. We have revised both the introduction and the literature review to establish a clearer and more cohesive narrative.

**Reviewer’s comment:** *The section argues convincingly that genuine participation remains limited, often devolving into engagement without influence. However, some statements could be strengthened with clearer empirical grounding.*

**Authors’ response:** We appreciate your insight and we have carefully revised the section to ensure all major statements are empirically supported.

**Reviewer’s comment:** *The section leans heavily on policy instruments, but not much on the organisational practice literature. You can draw on literature that examines internal organisational dynamics and decision making. This would strengthen the bridge between policy aspirations and everyday organisational realities, reinforcing the relevance of a decision-making tool designed for internal use.*

**Authors’ response:** We have expanded our literature review to incorporate additional empirical work on participation. However, we have deliberately chosen not to expand our scope into broader organizational practice literature. Because that field is exceptionally vast and often decoupled from the unique operational realities of museums and cultural organizations, we believe keeping a tight focus serves the manuscript best.

**Reviewer’s comment:** *Participatory workshops design and data:Overall, this section reads primarily as the methodological description of the study, outlining the organisational context (OrgC), the workshop protocol, and the data collection process. It is clear and detailed; however, several aspects could be strengthened to deepen its analytical depth and better connect it to the broader research aims.* *Clarify methodological positioning: The section could more explicitly signal how these workshops function within the overall research design. Currently, the narrative is descriptive, but the methodological rationale could be foregrounded more clearly.*

**Authors’ response:** Thank you for this suggestion. We have revised this section to signal more explicitly that the workshops functioned as an empirical testing ground to observe the protocol’s applicability.

**Reviewer’s comment:** *Strengthen the link between organisational context and analytical framing: The description of OrgC is informative, but it could connect more explicitly to why this organisational setting matters methodologically. Briefly connecting these characteristics to decision-making challenges would better justify the choice of case.*

**Authors’ response:** We have updated the opening paragraph of the sub-section *'From real problems to realistic scenarios’ *to provide a detailed justification of our choice of case studies.

**Reviewer’s comment:** *Show how the tasks connect to organisational inclusion: The workshop protocol is well-structured, but the feedback would be stronger if you could briefly explain why the three-task sequence was chosen, how it captures or disrupts existing organisational power patterns, how it supports more inclusive governance routines (especially in heritage settings where community voices are often marginalized etc).*

**Authors’ response:** Thank you very much for this comment. As explained in the introduction (par 4) of the paper: “By introducing an anonymous agenda proposal stage, we explore whether the patronising role of dominant members can be mitigated, thereby creating more equitable conditions for participation. Furthermore, we take into account that, while collective decisions are often facilitated, constrained and eventually shaped by processes intended to elicit and express individual preferences, an inherent challenge of every collective endeavour is that the final decision will necessarily deviate from each participant’s preferred outcome. Thus, by applying a protocol that combines voting with a post-voting brainstorming procedure, we examine how individual preferences may evolve during the course of decision-making, as participants learn about both their own and other’s preferences through observation, discussion and negotiation”.

**Reviewer’s comment:** *Describe how the qualitative data is analyzed in details*

**Authors’ response:** In the methodology section, we have added the following clarifying points: “To capture nuanced group dynamics, workshop discussions were recorded to generate qualitative data that contextualises and complements the analysis and interpretation of the final results.” (please see the sub-section, titled ‘ *Protocol’); *and   “Moreover, recordings from brainstorming and deliberation capture a rich layer of qualitative data on group dynamics that deepen our understanding of this process. Audio recordings were transcribed and provided vital context during subsequent analysis, shedding light into participants’ route of refining and re-building the “winner” solution into a final proposal - outcome, by common consensus” (please see the sub-section, titled ‘ *Implementation and data’*).

**Reviewer’s comment:** *Results***** *-* *Overall, the results are clearly articulated and provide compelling evidence. However, the section would be significantly strengthened by making more explicit how the three-task structure contributes to organisational inclusion and participatory governance.* *The results describe what happened in each workshop, but the manuscript would benefit from a clearer articulation of why Task A (individual proposals), Task B (voting), and Task C (deliberation) matter for inclusive governance. (how do they relate to each other)*

**Authors’ response:** Thank you very much – please note that this is analysed in detail in the discussion and conclusion of the paper. See, indicatively: “Our decision-making protocol introduces several key innovations. First, during the agenda-setting phase, proposals are anonymised to minimise biases associated with leader dominance or the identification of ideas with specific participants. Second, we employed a rigorous voting mechanism that integrated diverse attributes into the agenda, obtained endogenously from participants’ input. This design encourages deep deliberation, grounded in a shared understanding of attributes and a unifying vocabulary for democratic discussion, ultimately facilitating consensus. […] The application of the Borda Rule, as in many contexts, was sufficient to identify the collectively preferred solution. Nevertheless, this ‘final’ choice-by-voting did not remain intact during deliberation. In all cases, group discussion through real-time interaction and face-to-face verbal exchange yielded unanimously accepted solutions that voting alone could not achieve. The voting stage was not unnecessary; rather, it provided the foundation for individual participation, a democratically structured set of ideas, and a vocabulary of focal points for subsequent deliberation. The results of the workshops where the protocol was applied illustrate a process of cumulative learning and movement toward consensus. In fact, not only verbal exchange and negotiation during a brainstorming session, but even the mere observation of others’ preferred endpoints may lead group members to reconsider their own ideal solutions. The initial stage guarantees that preservation of individual proposals into the voting stage, shielded from strategic manipulation through simultaneous submission and proposers’ anonymity.”

**Reviewer’s comment:** *The results illustrate cumulative learning and movement toward consensus, but the implications for ongoing governance could be made more explicit.* **Authors’ response:** Please note that implications are discussed in the concluding remarks. In particular, see paragraph 7 “In terms of readiness and enabling conditions for the protocol’s implementation, successful application…”

**Reviewer’s comment: **Discussion and conclusion - *Overall, this section is empirically robust, synthesizes findings across the three cases, and clearly highlights both the methodological contribution of the decision-making protocol and its practical implications for cultural and heritage organisations.* *To strengthen the section, you may consider clarifying the through-line that connects your empirical findings to broader theoretical claims about participatory governance, organisational inclusion, and preference formation (referring back to the introduction and the literature review).*

**Authors’ response:** Thank you very much for this valuable suggestion. We have updated the concluding remarks to include our reflections on the literature. We have also expanded our discussion to highlight the protocol’s practical relevance to organisational practice.

**Reviewer’s comment:** *Some arguments would benefit from being brought into sharper dialogue with the heritage governance literature, and certain interpretive claims may need tighter framing or more explicit justification. For example, the metaphor of “fermentation” is compelling but may require more conceptual scaffolding.*

**Authors’ response:** Thank you for your guidance and encouraging remarks. We have elaborated on the fermentation metaphor in the *Discussion *section to deepen our analysis: “the observed dynamics during the voting and the consensual brainstorming sessions suggest that our decision-making protocol serves as a fermentation mechanism for individual and collective preferences, rather than a static platform for the transformation of unambiguous individual preferences into collective decisions.”   Furthermore, in the concluding remarks, we now comment the following: “The results of the workshops where the protocol was applied illustrate a process of cumulative learning and movement toward consensus. In fact, not only verbal exchange and negotiation during a brainstorming session, but even the mere observation of others’ preferred endpoints may lead group members to reconsider their own ideal solutions. This highlights the need for a foundational reconsideration of how collective decision-making has been studied across different strands of the relevant literature in order to account for the dynamics of learning about one’s own and others’ preferences throughout voting and deliberative processes.   In the context of cultural organisations, it also underscores that participatory management can also serve as a pathway for active learning, meaning-making and reflective observation (Simon, 2010; Black, 2018). Across the workshops reported here, final consensual outcomes frequently diverged from both initial proposals and the ranked leader. Rather than signalling aggregation failure, this pattern can be interpreted as preference learning: private proposal stages reduce conformity pressures; exposure to structured alternatives expands the option space; and discussion clarifies meanings and aligns choices with shared values. Recording reasons and trade-offs provides a defensible basis for organisational legitimacy.”
